# Microbial Communities Associated With Indigo Fermentation That Thrive in Anaerobic Alkaline Environments

**DOI:** 10.3389/fmicb.2018.02196

**Published:** 2018-09-18

**Authors:** Keiichi Aino, Kikue Hirota, Takahiro Okamoto, Zhihao Tu, Hidetoshi Matsuyama, Isao Yumoto

**Affiliations:** ^1^Bioproduction Research Institute, National Institute of Advanced Industrial Science and Technology, Sapporo, Japan; ^2^Department of Bioscience and Technology, School of Biological Science and Engineering, Tokai University, Hiratsuka-shi, Japan; ^3^Graduate School of Agriculture, Hokkaido University, Sapporo, Japan

**Keywords:** indigo fermentation, *Alkalibacterium*, *Amphibacillus*, *Polygonibacillus*, *Fermentibacillus*, *Paralkalibacillus*, *Anaerobacillus*

## Abstract

Indigo fermentation, which depends on the indigo-reducing action of microorganisms, has traditionally been performed to dye textiles blue in Asia as well as in Europe. This fermentation process is carried out by naturally occurring microbial communities and occurs under alkaline, anaerobic conditions. Therefore, there is uncertainty regarding the fermentation process, and many unknown microorganisms thrive in this unique fermentation environment. Until recently, there was limited information available on bacteria associated with this fermentation process. Indigo reduction normally occurs from 4 days to 2 weeks after initiation of fermentation. However, the changes in the microbiota that occur during the transition to an indigo-reducing state have not been elucidated. Here, the structural changes in the bacterial community were estimated by PCR-based methods. On the second day of fermentation, a large change in the redox potential occurred. On the fourth day, distinct substitution of the genus *Halomonas* with the aerotolerant genus *Amphibacillus* was observed, corresponding to marked changes in indigo reduction. Under open-air conditions, indigo reduction during the fermentation process continued for 6 months on average. The microbiota, including indigo-reducing bacteria, was continuously replaced with other microbial communities that consisted of other types of indigo-reducing bacteria. A stable state consisting mainly of the genus *Anaerobacillus* was also observed in a long-term fermentation sample. The stability of the microbiota, proportion of indigo-reducing microorganisms, and appropriate diversity and microbiota within the fluid may play key factors in the maintenance of a reducing state during long-term indigo fermentation. Although more than 10 species of indigo-reducing bacteria were identified, the reduction mechanism of indigo particle is riddle. It can be predicted that the mechanism involves electrons, as byproducts of metabolism, being discarded by analogs mechanisms reported in bacterial extracellular solid Fe^3+^ reduction under alkaline anaerobic condition.

## Introduction

Ancient human beings developed a number of environmentally friendly and renewable bioprocesses to benefit their communities. Hence, reexamining these procedures and understanding their scientific bases will aid the development of environmentally friendly procedures. In addition, these techniques can be modified for the development of highly sophisticated procedures. In this review, we discuss the molecular and microbiological bases for the traditional procedure for dyeing textiles and reconsider these procedures for the design of environmentally friendly bioprocesses.

Since ancient times, humans have dyed textiles with pigments, mainly plant pigments. Indigo is one of the oldest dyes and has been used since the Neolithic period (Clark et al., 1993). Textiles pigmented using indigo plants were discovered on an Egyptian mummy from 2500 BC (Balfour-Paul, 2000; Gilbert and Cooke, 2001). Recently, evidence for the earliest use of indigo, dating back to approximately 4000 BC, was obtained from Huaca Prieta, in contemporary Peru (Splitstoser et al., 2016). The polygonaceous Japanese indigo plant (*Polygonum tinctorium* Lour.) has been used in China, Korea, and Japan. Ryukyu-Ai (*Strobilanthes cusia*) has been used in the Ryukyu Islands (in contemporary Okinawa Prefecture), Japan. Indigofera (*Indigofera tinctoria* L. and *Indigofera suffruticosa* Mill.) and woad (*Isatis tinctoria* L.) have been employed in India and Europe (Hurry, 1930; Clark et al., 1993), respectively.

**FIGURE 1:**
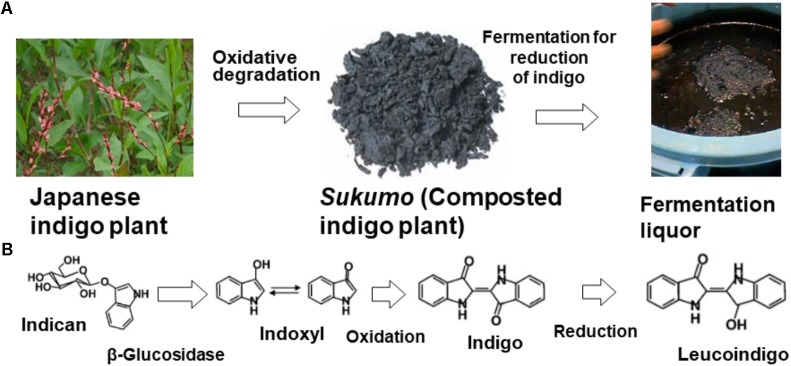
Scheme for indigo dyeing via the traditional fermentation method using indigo plant leaves **(A)** and the concomitant chemical changes in the dye **(B)**. The origin of the dye is indican, present in the Japanese indigo plant. Indican is transformed during the production of *sukumo*. Indigo is reduced by the action of indigo-reducing bacteria and is transformed to leucoindigo.

Several processing procedures for the preservation and transportation of indigo dye-containing plants have been used worldwide. Indigo can be extracted from indigo dye-containing plants with water and is processed by intrinsic enzymatic reactions carried out by β-glucosidase in chloroplasts (Minami et al., 1997; Song et al., 2010), which transforms indican (no color) to indoxyl (no color) and produces indigo dye via oxidation (when exposed to air). The original state of indigo dye is always indican (**Figure 1**). Therefore, transformation of indican to indoxyl is necessary for the production of indigo dye for dyeing textiles. The extracted indigo dye is oxidized by aeration, heated, and air dried or used to make a paste with lime hydrate (Ca(OH)_2_). The former method is popular in India. The latter method is popular in Okinawa Prefecture in Japan, the northeastern part of India (a mountainous region), southeast Asia, and southern China. Alternatively, indigo-containing plants are composted by microorganisms, and indican is transformed to indigo via the formation of indoxyl during this process (**Figure 1**). This procedure is performed in Japan, Europe, northeastern India, and West Africa. However, the composting procedure and plant material differ depending on the country.

Indigo extraction from indigo-containing leaves is quite an effective method for enrichment of indigo dye. However, if the collected indigo dye is heated to destroy microorganisms for preservation, direct fermentation might be difficult in the subsequent step of indigo reduction by microorganisms. The reducing power of the plant *Cassia tora* has been previously used in such a scenario. On the other hand, the composting procedure sustains microorganisms for subsequent indigo reduction (indigo → leucoindigo) (**Figure 1**). In Europe, a composting procedure that uses woad was developed in the Middle Ages. Harvested woad leaves are cut into very small pieces and gathered into a ball that fits in the palm of a hand. These prepared balls are then fermented for a short period under appropriate moisture conditions and dried completely to produce dried woad balls. Then, the balls are crushed, and the products are fermented over a long period via the addition of water to produce appropriate conditions for microbial activation, including an increase in the temperature to 55°C. After approximately 2 weeks of being subjected to microbial degradation, the “couched woad” is allowed to dry (Padden et al., 2000). Subsequently, the obtained product is transferred to the woad vat, which is maintained at 50°C, for a second fermentation to induce indigo reduction.

In Japan, a method for composting indigo-containing plants has been developed in Tokushima Prefecture, Shikoku, Japan (34°04′ N, 134°31′ E). The production of *sukumo*, the composted Japanese indigo plant, was developed not only for the preservation and transportation of indigo dye but also for enrichment of the indigo dye in the plant. In addition, the remaining microorganisms serve as inocula for the culture in next fermentation step, and the microorganisms present in *sukumo* can survive for at least 5 years. Furthermore, the remaining plant materials can be used as nutrient sources for microbial growth in the next fermentation step and may aid the attachment of the essential microorganisms to the debris.

Indigo is transformed to leucoindigo by the action of indigo-reducing bacteria (indigo → leucoindigo) (**Figure 1**). Although indigo is not soluble in water, leucoindigo is soluble in water. Therefore, leucoindigo penetrates textiles dipped in indigo fermentation fluid, followed by a brief exposure to air to oxidize leucoindigo (leucoindigo → indigo). Thus, indigo fixed in the textile. Traditional indigo fermentation is difficult to initiate, and long-term maintenance of the fermentation fluid in an indigo-reducing state (leucoindigo) is also challenging because the presence of the microorganisms depends on their natural occurrence and on appropriate maintenance procedures (i.e., stirring once a day, maintaining the pH, appropriately timing the feeding of the microorganisms), which requires much experience. Therefore, the fermentation procedure has been replaced with chemical reduction using sodium dithionite (Na_2_S_2_O_2_). However, the addition of Na_2_S_2_O_2_ produces environmentally unfavorable products, which leads problems in the disposal of the dye waste (Bechtold et al., 1993; Božič and Kokol, 2008). Sodium dithionite is ultimately oxidized to toxic derivatives such as sodium sulfate (Na_2_SO_4_), sulfite ions (${\text{SO}}_{3}^{2-}$), and thiosulfate (${\text{S}}_{2}{\text{O}}_{3}^{2-}$). When sodium dithionite is dumped into a water treatment system, these chemical damages the activated sludge due to its strong reducing power.

Thus, the development of conventional procedures, including management systems that do not involve the use of chemical reagents, is indispensable for the reemerging of the technique of indigo reduction by microorganisms. To achieve this goal, it may be helpful to identify the microorganisms responsible for indigo reduction and the mechanisms of indigo reduction during both the initial transitional changes and the stable state in the fermentation process. In addition, it is important to elucidate the maintenance mechanisms of the microbiota under open-air conditions and the mechanisms of deterioration toward the end of fermentation. Currently, craftspeople employ specific procedures that they have developed themselves. Therefore, there are likely many possible appropriate procedures. Analysis of many kinds of fermentation fluids in various fermentation stages is necessary for elucidation of the core mechanisms associated with the transitional changes in the microbiota during indigo fermentation.

## Traditional Procedures for Indigo Dyeing in Japan

The polygonaceous Japanese indigo plant harvested during summer is cut to a length of 1.5 cm. The following day, the obtained product is air dried and separated into leaves and stem, and the leaves are placed in a straw bag. At the beginning of September, the leaves are piled on an earthen floor (an indoor ground place; approximate size: 5 × 5 m) to a thickness of approximately 1 m and appropriately wetted to induce decomposition by microorganisms. Fermentation begins after approximately 4–5 days, and produces ammonia, which is identifiable by its odor, at a temperature of 70°C. Fermentation of the indigo leaves is promoted by appropriate regulation of the moisture content to maintain aerobic conditions and high temperatures by adjusting the turnover frequency and adding water; this process requires the technical skills of a trained and well-experienced craft person. This fermentation step is continuously performed through the end of December, and the product, referred to as *sukumo* (**Figure 1**), is then covered with a straw mat. The production of *sukumo* is difficult to manage. In addition, there are only a few craftspeople who can produce *sukumo*. Therefore, to maintain an environmentally friendly indigo-reducing procedure, alternative procedures for the traditional production of *sukumo* should be developed in the near future.

**FIGURE 2:**
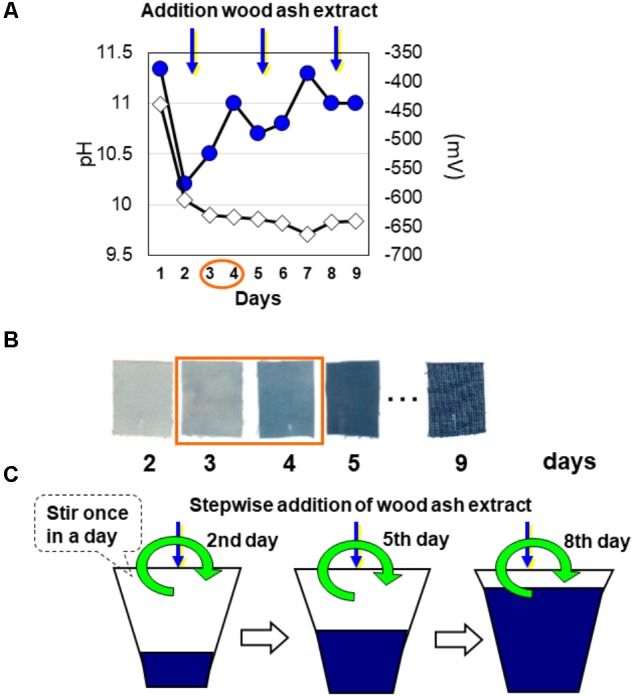
Changes in abiotic parameters in the preparation of indigo fermentation systems and the preparation scheme. The measured parameters were pH (filled circles) and ORP (open diamond) **(A)**. The textile dyeing intensity **(B)** was estimated by dipping a strip of textile. Scheme of preparation for initiation via stepwise addition of wood ash extract **(C)** to the fermentation liquor used for indigo dyeing during the initial incubation period. The indigo fermentation batch was prepared in the laboratory. This figure was reproduced from a figure in Aino et al. (2011) with some modifications. This reproduction was permitted by Oxford University Press.

In the liquid fermentation step for reduction of the indigo contained in *sukumo*, the insoluble oxidized state of indigo in *sukumo* is solubilized by indigo-reducing microorganisms (**Figure 1**). First, *sukumo* is wetted with hot wood ash extract (80°C) and mixed well in a container. The obtained clay-like product is subsequently kneaded well, added to a small amount of Japanese rice wine, and allowed to settle overnight at room temperature. Next, hot wood ash extract is added at up to one-third of the final volume. After indigo reduction occurs, fermentation liquid is added at up to two-thirds of the final volume. The effective reduction of indigo is then verified, and fermentation fluid is added to obtain the final volume (**Figure 2C**). The resulting indigo-reduced fermentation mixture is maintained at a pH greater than 10.3–10.5 by adding lime hydrate (Ca(OH)_2_) and is stirred once daily. Based on the dyeing intensity (determined by checking the staining of cotton cloth), wheat bran is added as a substrate for the indigo-reducing microorganisms.

Indigo reduction is initiated by spontaneously occurring microbes that probably originate from the *sukumo*. Therefore, the period between the initiation of the fermentation and the appearance of indigo (from 3 to 4 days to greater than 2–3 weeks) varies depending on the quality of the *sukumo* and the preparation procedure. This uncertainty in the reduction leads many people to add chemical reducing agents. The indigo-reducing state during liquid fermentation is sustained by the microbiota. Therefore, if the microbiota in the fermentation mixture becomes imbalanced, the indigo-reducing state of the fermentation mixture is not maintained, which is why long-term fermentation for indigo dyeing is difficult to maintain. The liquid fermentation period changes depending on the capacity of the fermentation vat, the initial microbiota, the shape of the container, the maintenance procedure, and the frequency of dyeing. The indigo-reducing state is maintained for an average of approximately 6 months under conditions that introduce the risk of contamination by unfavorable microorganisms, especially during the dyeing occasion (i.e., contaminants introduced by the dipping of textiles and by hands). Although the fermentation mixture has a high pH and presents unfavorable conditions for ordinary microorganisms to thrive, neutralophilic microorganisms can survive under such conditions due to the existence of localized niches that exhibit lower pH values than the bulk environment. Such localized niches could be generated by acid-producing bacteria. In fact, some indigo-reducing bacteria, such as *Alkalibacterium* spp., produce lactic acid (Yumoto et al., 2014). Therefore, it is considered that stirring once a day is important to prevent the spread of small, localized, neutral niches in the fermentation fluid.

## An Early Study on Indigo-Reducing Bacteria

Identification and application of indigo-reducing bacteria are essential for the improvement of indigo fermentation. The first indigo-reducing bacterium was discovered by Takahara and Tanabe (1960). Based on phenotypic characterization, they identified the isolate as belonging to the genus *Bacillus*. Given its novel characteristics, the isolate was considered a new species, which they named ‘*Bacillus alkaliphiles*.’ The cell size of this bacterium is 0.9–1.0 × 2.5–3.5 μm, and it occurs both singly and in pairs. This motile, Gram-negative bacterium grows at a high pH (e.g., pH 10) and exhibits a strong ability to reduce the redox potential of indigo fermentation fluid. The optimum pH for the growth of this bacterium is 10–11.5. This species grows at temperatures between 10 and 50°C, with an optimum temperature of 30°C. However, the growth rate of this bacterium decreased at temperatures greater than 36°C. This bacterium is a facultative anaerobe and requires a growth factor that consists of seven amino acids. This species produces spores, is catalase positive, and hydrolyzed gelatin and starch. Since Takahara and Tanabe did not deposit the ‘*B. alkaliphiles*’ strain in a culture collection, it is impossible to compare directly ‘*B. alkaliphiles*’ with recently isolated indigo-reducing bacteria.

## Studies on Woad Dye Fermentation Vats

In 1998, the indigo-reducing *Clostridium isatidis* was isolated from a couched woad vat prepared by a traditional medieval European procedure (Padden et al., 1998a,b, 2000). This bacterium was isolated in a medium at pH 9.0 that was incubated at 47°C. The cell size of this species is 0.3–0.6 × 1.8–9.1 μm. *C. isatidis* is Gram positive and aerotolerant but strictly anaerobic for growth. This moderate thermophilic bacterium occurs singly, in pairs, or in chains; and produces terminal endospores. The pH range for the growth of this bacterium is 5.9–9.9, with an optimum pH of 7.2 (at 50°C). The temperature range for the growth of this bacterium is 30–55°C, with an optimum temperature of 49–52°C (at pH 7.2). This strain produces a large amount of gas, which consists of carbon dioxide and hydrogen. This strain also produces acids, mainly acetic, lactic, and formic acids. In addition, three more strains to remove oxygen were identified. Two strains, *Bacillus pallidus* and *Ureibacillus thermosphaericus*, which have already mentioned in relation to the couching process and *Bacillus thermoamylovorans* probably consume oxygen through respiration (Cardon, 2007). The fermentation conditions for this strain differ from those for the method using *sukumo* in terms of pH and temperature. *C. isatidis* is assumed to be a specific indigo-reducing bacterium that is present in couched woad. Although the pH level during fermentation with this strain is not sufficiently stringent to exclude neutralophilic bacteria, the fermentation temperature is higher than that of the Japanese procedure. Therefore, it can be assumed that the pH combined with the temperature is stringent enough to exclude commonly existing bacteria. The microbiota of the couched woad fermentation fluid differs from that of the Japanese method that uses *sukumo* as will be discussed below.

The indigo reduction mechanism was studied using *C. isatidis* (Nicholson and John, 2005). The diameter of the indigo particles was at least 50 times that of the bacterial cells (Compton et al., 2000). Therefore, it can be presumed that indigo-reducing bacteria solubilize solid matter that is much larger than the bacterial cells for reduction. Comparative studies of the indigo-reducing *C*. *isatidis* and four other *Clostridium* species showed that the indigo-reducing ability of this strain was not shared by the other species. The culture supernatant from *C*. *isatidis* decreased the indigo particle size to one-tenth of the initial diameter, which was not observed with the other species. An electron mediator, anthraquinone-2,6-disulfonic acid (AQDA), stimulated indigo reduction by *C*. *isatidis*. *C*. *isatidis* exhibited a greater ability to reduce ambient redox potential than the other strains used in this study. The redox potential of *C. isatidis* culture was −600 mV, which was 100 mV lower than the redox potentials of the other four *Clostridium* spp. Although the authors mentioned that quinone probably acts by modifying the surfaces of the bacteria or indigo particles, it is possible that quinone acts as an electron mediator in the reduction of indigo because the addition of quinone accelerates indigo reduction. The decrease in indigo particle size caused by the culture supernatant of *C*. *isatidis* suggested that the electron-retaining quinone from woad reduced the indigo particle. Thus, as an electron mediator, quinone is considered to be very important for indigo reduction in woad dye vats. On the other hand, direct electron transfer between *C*. *isatidis* cells and carbon electrodes has been demonstrated (Compton et al., 2000). Although the report indicated that *C*. *isatidis* could transfer electrons to the solid material, it was not determined whether the bacterium can reduce indigo particles directly.

Two broth media, containing either yeast extract (extracted from baker’s yeast; 30 g L^−1^) or corn steep liquor (CSL; 10 g L^−1^), were used for their capacity to sustain the growth and reducing activity of *C*. *isatidis* (Osimani et al., 2012). A relatively high viable cell count and low oxidation-reduction potential (ORP) value were observed in the CSL-containing broth. Subsequently, in order to examine sustainability, CSL broth treated with 140 g L^−1^ woad powder and 2.4 g L^−1^ indigo dye under sterilized conditions was fermented under anaerobic or microaerobic conditions. In all the fermentation batches, sufficiently low ORP values for reducing indigo were attained within 24 h and were maintained for up to 9 days. However, the total counts of vegetative cells and spores were higher under anaerobic conditions. In addition, rapid indigo dye reduction was observed under strictly anaerobic conditions. This observation indicates the superiority of the original woad system compared to the indigo dye system for indigo reduction in CSL broth, as the original system did not require the introduction of N_2_. This suggest that microbiota containing in the original woad system have a role to accelerate in the fermentation vat under atmospheric condition.

The microbiota of woad vat fermentation liquor aged for 12 months has been examined via PCR-DGGE (denaturing gradient gel electrophoresis) and pyrosequencing (Milanović et al., 2017). Bands corresponding to *Paenibacillus lactis* (98–99% identity) and *B. thermoamylovorans* (99%) were frequently observed in these assays. Bands corresponding to *Bacillus pumilus* (92–94%), *Sporosarcina koreensis* (99%), and *Bacillus licheniformis* (99%) were also observed. These bacterial members are rarely observed in the Japanese procedure that uses *sukumo*. This indicates that the fermentation conditions of this method have a lower pH and higher temperature than those of the *sukumo*-using method.

By pyrosequencing analysis, *Clostridium ultunense*, *Alcaligenes faecalis*, and *Tissierella* spp. were observed to be the predominant members of the dyeing fluid (Milanović et al., 2017). *Virgibacillus pantothenticus* and *Virgibacillus* spp. were also detected as minor constituents. Unidentified Bacillaceae and Clostridia, including moderately thermophilic bacteria, lactic acid bacteria, and photosynthetic bacteria, were observed among the subdominant components. These results suggest that the reported indigo-reducing bacteria are not major members of this fermentation fluid. However, all the indigo-reducing bacteria have not been identified yet. Therefore, there is a possibility that some of the detected members are indigo-reducing bacteria. Furthermore, there is a possibility that this pyrosequencing analysis could not detect indigo-reducing bacteria because pyrosequencing analysis results can vary depending on bacterial community structure, sequencing technology, and PCR bias (Aird et al., 2011; Klindworth et al., 2012; Lee et al., 2012; Luo et al., 2012; Pinto and Raskin, 2012; Wu et al., 2012; O’Donnell et al., 2016).

## Indigo-Reducing Enzymes

**FIGURE 3:**
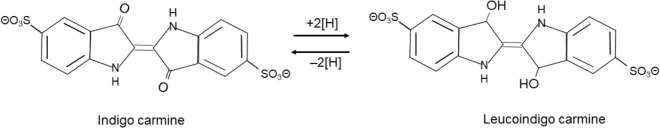
Chemical structure of indigo carmine and its reduction/oxidation.

An indigo-carmine (**Figure 3**)-reducing enzyme from a *Bacillus cohnii* strain that was isolated from an indigo fermentation system has been purified and characterized (Nii et al., 2006). The enzyme was purified from disrupted cells, and the enzymatic activity was determined using NADH and indigo carmine as the electron donor and acceptor, respectively. The optimum pH for the activity is pH 7.5, and the enzyme is stable at pH 3.5–9.5. The optimum temperature for the activity is 30°C, and the enzyme is stable at temperatures up to 30°C. The activity increases substantially when 2,6-dichlorophenol-indophenol is used as the electron acceptor instead of indigo carmine. The molecular mass was determined to be 74 kDa by gel filtration. The reported enzyme is considered to be a kind of azoreductase. A few azoreductases have been reported in *Bacillus* spp. (e.g., *Bacillus* sp. and *Bacillus cereus*) (Ooi et al., 2007; Pricelius et al., 2007). These azoreductases are thought to react with soluble substrates such as indigo carmine. It is considered that it is unlikely that these azoreductase-like enzymes react directly with solid substrates that have diameters at least 50 times larger than those of the bacterial cells. However, an azoreductase which oxidized NADH in presence of indigo was reported by Suzuki et al. (2018). Large indigo particles may also react with electron mediators such as quinone or by electrically conductive pilus-like “nanowires.” However, it has been considered that azoreductases from *Bacillus* spp. will contribute to the maintenance of indigo in the reduced state.

## Extracellular Reduction in Alkaliphiles

**FIGURE 4:**
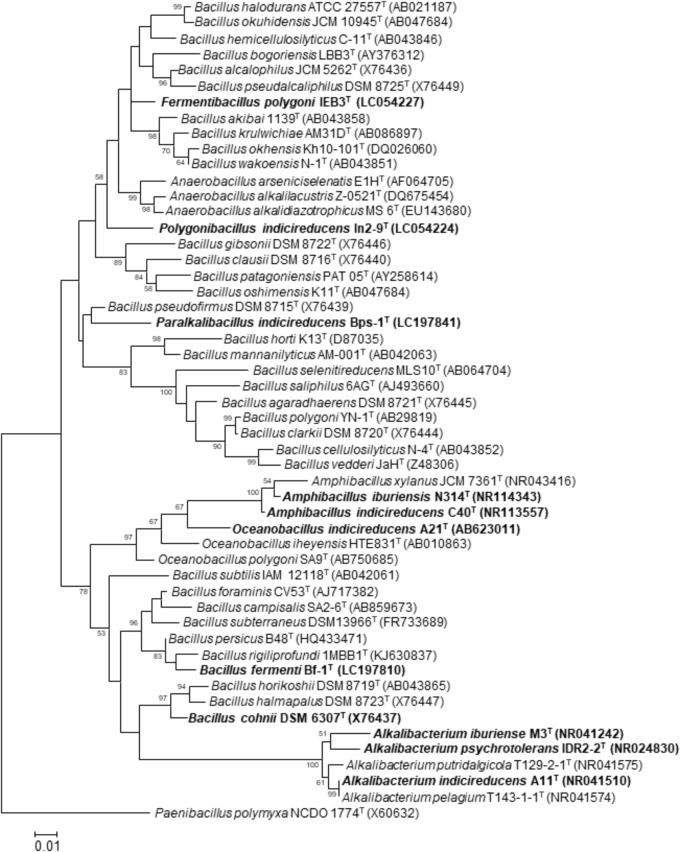
Maximum-likelihood phylogenetic tree derived from 16S rRNA gene sequences, showing the phylogenetic positions of the isolated indigo-reducing bacteria. To construct the phylogenetic tree, the sequences were aligned with the sequences of neighboring species, and the consensus sequence was determined by CLUSTAL W (Thompson et al., 1994). The evolutionary history was inferred by using the maximum-likelihood method (Guindon and Gascuel, 2003) in MEGA 7 (Kumar et al., 2016). The evolutionary distance matrix was calculated by using Kimura’s two-parameter model (Kimura, 1980). Bold letters indicate the indigo-reducing bacteria. Although *B. cohnii* DSM 6307^T^ was not one of our isolates, strains belonging to this species that reduced indigo were isolated from indigo fermentation fluid. Bootstrap percentages (based on 1000 replicates) >50% are shown at branch points. Scale bars = 0.01 substitutions per nucleotide position.

If an alkaliphile that is present in indigo dye vats has the ability to reduce extracellular substances, it is possible that the microorganism has the ability to reduce indigo particles. Ma et al. (2012) isolated an alkaliphilic *Bacillus*, namely, *Bacillus pseudofirmus* MC02, that can transfer electrons to anthraquinone-2,6-disulfonate (AQDS), humic acids (HAs), and Fe(III) oxides as representative electron acceptors. This strain could effectively perform Fe(III) oxide reduction coupled with sucrose fermentation when AQDS was added as an electron mediator. In addition, this bacterium can decolorize azo dyes in alkaline conditions. Although it has not been determined whether *B*. *pseudofirmus* MC02 can reduce water-insoluble indigo, this bacterium occupies the same branch as the indigo-reducing bacterium *Paralkalibacillus indicireducens* (**Figure 4**). *Anaerobacillus arsenicoselenatis* and *Anaerobranca californiensis* have been reported as exhibiting Fe(III) reduction (Blum et al., 1998; Gorlenko et al., 2004). As described below, *Anaerobacillus* spp. and *Anaerobranca* spp. are frequently observed in indigo fermentation vats. While the *Anaerobacillus* and *Anaerobranca* strains mentioned above were examined using soluble Fe^3+^ as an electron accepter, *B*. *pseudofirmus* MC02 could reduce both soluble Fe^3+^ and solid-phase Fe(III) oxides.

## Effect of Redox Potential

Addition of AQDA (0.003–0.01%) stimulates indigo reduction. However, it is considered that AQDA does not contribute directly to reduction of the redox potential in bacterial cultures in that study, due to its higher midpoint redox potential than that of indigo (Nicholson and John, 2005). The midpoint redox potential (*E’*^0^) of AQDS is −184 mV at pH 7 (Clark, 1960) or −290 mV at pH 9 (Chatterjee et al., 1998). The midpoint potential of indigo is difficult to estimate due to the insolubility of the oxidized form of this compound. An indicative value of −474 mV [versus a saturated calomel electrode (SCE)] in water at 50°C was determined in the presence of solid indigo (Vickerstaff, 1954). However, an even lower redox potential (−600 mV) is considered to be required for indigo reduction in industrial practice (Bechtold et al., 1993). Although theoretical electron transfer from AQDS is difficult, the finding of acceleration of indigo reduction by AQDS indicates that the difference in redox potential between AQDA and an indigo particle at the surfaces of the indigo particle may be different from the bulk redox potential that is necessary to reduce indigo in the absence of electron mediators.

## Indigo-Reducing Bacteria Belonging to the Genus *Alkalibacterium*

We attempted to isolate indigo-reducing bacteria from an enrichment culture using a conventional broth medium (pH 10) under anaerobic conditions with fermentation fluid obtained from the craft center. Commercially available indigo powder, which is insoluble in water, was used as an indicator of indigo reduction. This enrichment culture was repeated five times and then transferred to conventional agar medium. By this procedure, we isolated the strain IDR2-2^T^, which can reduce indigo (Yumoto et al., 2004). This strain was identified as a member of the genus *Alkalibacterium* by 16S rRNA gene sequence analysis (**Figure 4**). This strain was identified as a new species because it differed from the only species of this genus that had been discovered up to that point, namely, *Alkalibacterium olivapovliticus*. Therefore, we named this strain *Alkalibacterium psychrotolerans* (Yumoto et al., 2004; **Table 1**). This strain grew equally well under both aerobic and anaerobic conditions and produced L-lactic acid. In addition to *Alkali. psychrotolerans*, *Alkalibacterium iburiense* (Nakajima et al., 2005; **Table 1**) and *Alkalibacterium indicireducens* (Yumoto et al., 2008; **Table 1**) were subsequently isolated and characterized (**Figure 4**). All the strains of *Alkali*. *psychrotolerans* and *Alkali*. *iburiense* were isolated from fermentation fluid obtained from Date City, Hokkaido, Japan, whereas *Alkali*. *indicireducens* was isolated from an indigo fermentation fluid sample obtained from Tokushima Prefecture, Shikoku, Japan. Although these species have similar characteristics to *A*. *psychrotolerans*, they differ from one another. For example, *A*. *psychrotolerans* grows faster than the other two species. In addition, *Alkali. psychrotolerans* and *Alkali*. *iburiense* produce acid from a number of carbohydrates, whereas *Alkali*. *indicireducens* did not produce acid from several tested carbohydrates. Although the preparation and maintenance procedures differed from the Japanese procedure, *Alkalibacterium* sp. has been isolated from a natural fermentation system used for 6 years for dyeing cotton textiles in Korea (Park et al., 2012).

**Table 1:** Characteristics of indigo-reducing bacteria found in indigo fermentation fluid.

	1	2	3	4	5	6	7	8	9	10	11
Cell shape	Rod	Rod	Rod	Rod	Rod	Rod	Rod	Rod	Rod	Rod	Rod
Gram stain	+	+	+	+	+	+	+	+	+	+	+
Cell size (μm)	0.4–0.9 × 0.7–3.1	0.3–0.4 × 1.7–3.0	0.4–1.2 × 1.7–3.7	0.3–0.5 × 1.0–3.0	0.3–0.4 × 1.7–3.0	0.4–0.9 × 1.7–2.6	0.6–1.0 × 1.3–4.5	0.7–0.8 × 2.0–6.4	0.5–0.8 × 1.1–2.0	0.8–1.2 × 2.2–3.8	ND
Aerotolerant anaerobic^∗^	Y	Y	Y	Y	Y	N	N	N	N	N	N
Facultatively anaerobic^∗^	N	N	N	N	N	Y	Y	Y	Y	Y	Y
Oxidase	−	−	−	−	−	+	+	+	+	+	+
Catalase	−	−	−	−	−	+	+	+	+	+	+
Spore location^§^	No spore	No spore	No spore	T	T	STC	STC	STC	T	STC	T
Flagella^¶^	Per	Per	Per	Per	Per	Per	Pair of subpolar	Per	Pair of subpolar and center side	Pair of subpolar	Per
Growth pH range	9–12	9–12	9–12.3	9–12	8–9.1^‡^	7–12	7.5–12	8–12	8–11	8–12	ND
Optimum growth pH	9.5–10.5	9.5–10.5	9.5–11.5	10	8.9–9.0^‡^	10	9 or 10	10	9–10	9–10	ND
Growth NaCl concentration range (%)	0–17	0 to 14–16	0 to 14–15	0–7	0–7	0–10	0–5 or 0–10	0–10	0–10	0–7	ND
Optimum growth NaCl concentration (%)	2–12	3–13	1–11	0–1	0–3	1–11	0	3	7	0–3	ND
Growth temperature range (°C)	5–45	5–45	15–40	17–39	26–39	18–48	12–40	10–45	18–40	15–45	10–47
Optimum growth temperature (°C)	34	30–37	20–30	35	36	39	30–33	35–37	33	33–40	ND
Isoprenoid quinone	No quinone	No quinone	No quinone	No quinone	No quinone	No quinone	MK-7	MK-6	MK-7	MK-7	MK-7
DNA G+C content (mol%)	40.6	42.6–43.2	47.0–47.8	37.5–37.7	38.4	39.7	39.0	39.4	40.3	41.7	33.5–35.0
Hydrolysis of cellulose and xylan	+	+	+	+	+	−	−	−	−	−	ND
Origin	Fermentation fluid prepared in a craft center	Fermentation fluid prepared in a craft center	Fermentation fluid prepared in a craft center	10-month-old aged fermentation fluid obtained from a craft center	10-month-old aged fermentation fluid obtained from a craft center	4th-day fermentation fluid prepared in a laboratory	Fermentation fluid prepared in a laboratory	Aged fermentation fluid obtained from a craft center	Aged fermentation fluid obtained from a craft center	Aged fermentation fluid obtained from a craft center	Aged fermentation fluid obtained from a craft center

*Strains: 1, Alkalibacterium psychrotolerans; 2, Alkalibacterium iburiense; 3, Alkalibacterium indicireducens; 4, Amphibacillus indicireducens; 5, Amphibacillus iburiense; 6, Oceanobacillus indicireducens; 7, Fermentibacillus polygoni; 8, Polygonibacillus indicireducens; 9, Paralkalibacillus indicireducens; 10, Bacillus fermenti; 11, Bacillus cohnii. Data for the strains were obtained from Hirota et al. (2013a; 2013b; 2013c; 2016a; 2016b; 2017; 2018), Nakajima et al. (2005); Spanka and Fritze (1993), Yumoto et al. (2004, 2008). +, positive; –, negative; ND, not determined ^∗^Y, yes; N, no. **^¶^** Per, peritrichous fhagellation. **^§^** T, terminal; STC, subterminal to central; C, central; ST, subterminal. ^‡^These pH values are based on time, while other descriptions are based on the initial pH of the medium.*

## Transitional Change in the Early Phase of Indigo Fermentation

Initiation of indigo reduction is expected to be associated with transitional changes in the microbiota in indigo fermentation vats under anaerobic alkaline conditions. Changes in the composition of the microbiota upon initiation of indigo reduction were examined via PCR-DGGE (Aino et al., 2011). Wood ash extract was added to *sukumo* to make a paste (1st day), and the 1st, 2nd, and 3rd volume expansions were performed on the 2nd, 5th, and 8th days. For each expansion, one-third of the total volume of wood ash extract was added to the fermentation system (**Figure 2C**). The addition of wood ash extract may induce a dramatic change in the microbiota, concomitant with the change in the environment. In fact, distinct changes in the PCR-DGGE banding pattern were observed from the 1st to 2nd; 2nd to 3rd; 5th to 6th; and 8th to 9th days (66.9, 39.1, 32.1, and 24.1%, respectively). The change in the banding pattern of the microbiota between the 1st and 2nd days (66.9%) was higher than that in the other periods within the first 9 days after preparation of the fermentation mixture. During the initiation of fermentation (1st to 2nd day), the ORP and pH changed dramatically, from −440 to −640 mV and from 11.3 to 10.2, respectively (**Figure 2A**). This substantial change in ORP may be attributed to the consumption of oxygen by aerobic microorganisms and the extracellular reducing activity of the excised microorganisms. The large pH change may be attributed to the byproducts of anaerobic metabolism. The corresponding PCR-DGGE bands that were enhanced were assigned to *Bacillus* sp. (similarity to *Bacillus firmus*: 95%) and *Amphibacillus* spp. Bands corresponding to *Halomonas* sp. and *Bacillus* sp. (similarity to *Bacillus cellulosilyticus*: 98%) were also observed. Therefore, *Bacillus* sp. (similarity to *B. firmus*: 95%) and *Amphibacillus* sp. (similarity to *Amphibacillus xylanus*: 99.2%) may play an important role in the dramatic change observed in the early phase of fermentation. Between the 3rd and 4th days from the initiation of fermentation, characteristics of indigo reduction were observed (**Figure 2B**). During this period, a relatively large change in the microbiota occurred (dissimilarity value, 28.2%). Bands attributed to *Amphibacillus* sp. (similarity to *A. xylanus*: 99.2), *Bacillus* sp. (similarity to *B. cellulosilyticus*: 96.4%), and *Corynebacterium* sp. [similarity to *Corynebacterium* sp. BBP21 (DQ337522): 99.2%] increased in intensity. The bacteria to which these bands were attributed may play significant roles in the transition to indigo reduction.

Since a change in the intensity of indigo dyeing was observed (**Figure 2B**), analysis of the microbiota using a 16S rRNA gene clone library was performed (Aino et al., 2011). On the 3rd day, the major constituents at the genus level were *Halomonas* (54%) and *Tissierella* (14%), whereas the genera *Tissierella* (35%), *Amphibacillus* (19%), and *Paenibacillus* (11%) were observed on the 4th day. The proportion of *Halomonas* dropped to 8% by the 4th day. It can be concluded that the large decrease in the proportion of *Halomonas* (54% → 8%) and the increase in the proportion of the indigo-reducing genus *Amphibacillus* (not detected → 19%) led to a dramatic change in the dyeing intensity. Prior to this study, it was thought that *Alkalibacterium* spp. were the main microbes responsible for indigo reduction. We conclude that there are functional redundancies in indigo reduction within the microbiota.

The same approach was applied to 10-month-old fermentation products to understand how the microbiota behaves under long-term fermentation. The fermentation fluid was obtained from the craft center. Although fermentation had been maintained for a long period, the microbiota was much simpler than expected. The major members identified belonged to the genera *Amphibacillus* (35%), *Alkalibacterium* (18%), *Tissierella* (18%), and *Alcaligenes* (13%), which indicates that the major members were indigo-reducing genera and that the diversity of the microbiota was relatively low. The observed microbiota is an example of the long-term-maintained fermentation fluid, and this information could contribute to understand microbial communities of the long-term fermentation.

## Indigo-Reducing Bacteria Belonging to the Genera *Amphibacillus* and *Oceanobacillus*

During a trial aimed at the isolation of indigo-reducing bacteria concomitant with the above analysis of the microbiota from fermentation fluid that we prepared in our laboratory and aged (10-month-old) fermentation fluid obtained from the craft center in Date City, Hokkaido, Japan, using a medium containing 0.2% indigo carmine, indigo-reducing *Oceanobacillus indicireducens* (strain A21^T^; **Table 1**) and *Amphibacillus* spp. were isolated (Aino et al., 2011). Strain A21^T^ was isolated from the fermentation liquor on the 4th day after the initiation of fermentation (the day when indigo reduction was initiated). 16S rRNA gene sequence analysis and the phylogenetic tree based on the sequence indicated that strain A21^T^ belonged to the genus *Oceanobacillus* (**Figure 4**). According to the polyphasic taxonomic approach, the bacterium was identified as a new species of *Oceanobacillus*, with the proposed name *O*. *indicireducens* (Hirota et al., 2013b). Surprisingly, this bacterium exhibits aerobic metabolism even though it lacks isoprenoid quinones. This characteristic has been observed in only strain A21 among the members of *Oceanobacillus*. One of the reasons for this peculiar characteristic is probably that the strain was isolated from an anaerobic alkaline environment from which no other *Oceanobacillus* spp. have been isolated.

Two indigo-reducing obligately alkaliphilic strains, namely, C40^T^ and N214, were isolated from a 10-month-old sample obtained from the craft center in Date City in Hokkaido, Japan (Aino et al., 2011). Strain C40^T^ exhibited stronger indigo-reducing activity than a strain of *Alkali. indicireducens* and other strains belonging to the genus *Alkalibacterium* within 6 days from the beginning of incubation. 16S rRNA gene sequence analysis and the phylogenetic tree based on the sequence indicated that strains C40^T^ and N214 belonged to the genus *Amphibacillus* (strain C40^T^ in **Figure 4**). Based on the polyphasic approach, the two strains were considered to belong to a new species, for which the name *Amphibacillus indicireducens* sp. nov. was proposed (Hirota et al., 2013a; **Table 1**). Strain N314^T^ was isolated from the same sample. However, this strain exhibited a different phylogenetic position from that of *Amphi. indicireducens* and other reported *Amphibacillus* spp. (**Figure 4**). Therefore, the name *Amphibacillus iburiensis* sp. nov. was proposed for this strain (Hirota et al., 2013c; **Table 1**). The species exhibited similar characteristics to *Amphi. indicireducens*. This strain also grew on media with an adjusted pH of 8–12. However, the strain was able to change the pH of the medium by producing acid, and growth was initiated at pH 8.9–9.1. Although these growth characteristics may be observed in other indigo-reducing bacteria, we detected these characteristics by monitoring the transition of the medium with strain N314^T^. In addition to *Alkalibacterium* spp., *Amphibacillus* spp. also appeared to play important roles in the reduction of indigo in many cases. Species of these genera are able to hydrolyze xylan and cellulose (**Table 1**). This characteristic may explain why wheat bran has long been used for the maintenance of indigo fermentation fluid.

## *Fermentibacillus Polygoni*, an Indigo-Reducing Bacterium Belonging to a Novel Genus and Species

Although Takahara and Tanabe (1960) isolated an indigo-reducing bacterium belonging to the genus *Bacillus*, as described above, we were unable to detect such a bacterium in our trials. The *Bacillus* sp. isolated by Takahara and Tanabe required a peptide for growth. The peptide was derived from a component of the fermentation liquor. Therefore, during the trial conducted to isolate indigo-reducing bacteria from fermentation fluid prepared in our laboratory, we attempted to isolate indigo-reducing *Bacillus* species using a medium that contained indigo fermentation liquor. An aliquot of the sample was inoculated onto indigo fermentation liquor agar (IFLA) medium, which consisted of only indigo fermentation liquor, 1% Na_2_CO_3_, and 1.5% agar. The agar plate was incubated at 27°C for 1 week, and 29 colonies were isolated. Two strains, namely, IEB3^T^ and IEB4, were selected on the basis of the high similarities of their 16S rRNA gene sequences with those of *Bacillus* species and low similarities with those of other valid reported species. The phylogenetic tree constructed from the sequences of these strains and those of related taxa showed that strains IEB3^T^ and IEB4 occupied a distinct position from the members of the genus *Bacillus* (strain IEB3^T^ in **Figure 4**). In addition to their distinct phylogenetic position, these strains were distinct from neighboring species or genera in terms of flagellum and spore morphologies. Therefore, we considered these strains to represent a novel species within a novel genus, and the name *Fermentibacillus polygoni* gen. nov., sp. nov. was proposed for this species (Hirota et al., 2016a; **Table 1**). Although the isolates were obtained from IFLA medium, this species did not require indigo fermentation liquid for growth. This isolate was the first example of a novel species within a novel genus isolated from indigo fermentation. New methods for the isolation of constituent bacteria from indigo fermentation systems will increase the ease of isolating undiscovered microorganisms. Media that contain low nutrient concentrations will be useful for the isolation of undiscovered microorganisms associated with indigo fermentation (Janssen and Yates, 2002).

## Changes in the Microbiota in Aged Indigo Fermentation

**FIGURE 5:**
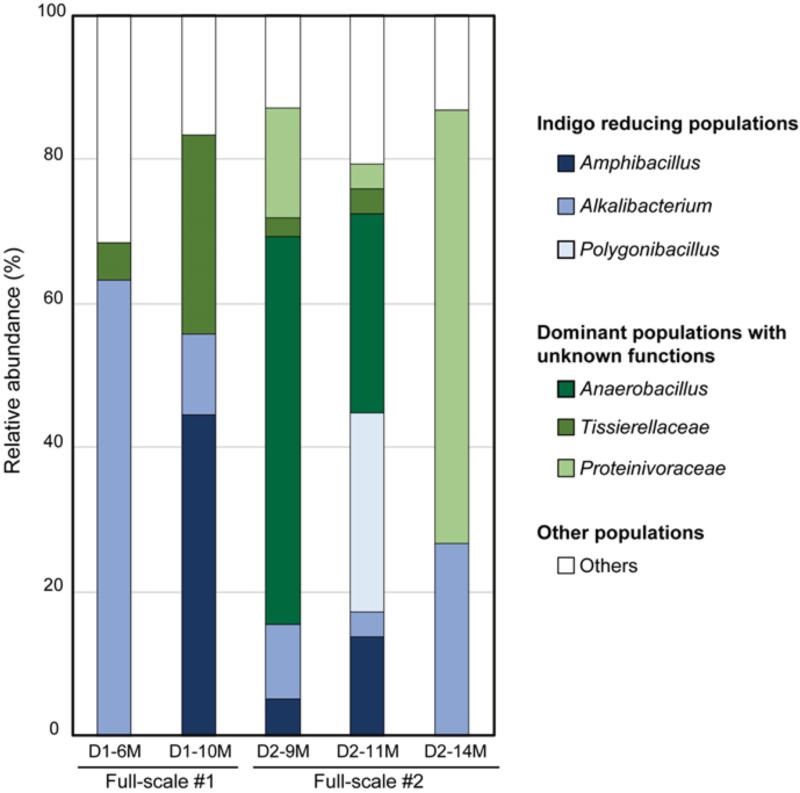
Changes in the bacterial community of the indigo fermentation fluid in aged phases (D1-6M, D1-10M, D2-9M, D2-11M, and D2-14M). The relative abundance of each taxon is shown. D1-6M, 1st-batch sample, aged 6 months, obtained from Date City (42°42′ N, 140°42′g E); D1-10M, 1st-batch sample, aged 10 months, obtained from Date City; D2-9M, 2nd-batch sample aged 9 months, obtained from Date City; D2-11M, 2nd-batch sample, aged 11 months, obtained from Date City; D2-14M, 2nd-batch sample, aged 14 months, obtained from Date City. This figure was cited from a figure in Okamoto et al. (2017) with some modifications. This citation was permitted by Springer-Nature.

Indigo reduction occurs via fermentation performed by naturally occurring microorganisms. This process continues under open-air conditions, and there are many chances for contamination. Indigo fermentation occurs under anaerobic and high-pH conditions. Although such conditions are far from the conditions under which ordinary microorganisms thrive, there are many microorganisms that produce acids in the fermentation fluid. It is presumed that there are microenvironments that have lower pH values than that of the bulk phase in the debris at the bottom of the fermentation fluid. Localized low-pH niches could develop if the liquid is not mixed sufficiently by daily stirring. Thus, although indigo fermentation fluid is a harsh environment for aerobic neutralophiles, there is opportunity for neutralophiles to propagate in this environment. Ordinary indigo fermentation maintains an indigo-reducing state for 6 months on average, and often for longer than 6 months. Hence, there must be mechanisms by which the bacteria involved in fermentation maintain the indigo-reducing state. Identification of the maintenance mechanism underlying long-term indigo fermentation could lead to the development of a novel, long-term, unsterilized bioprocess in the future. To determine the microbiological basis of the maintenance of indigo fermentation, we examined the microbiota in fermentation fluids maintained for more than 6 months (Okamoto et al., 2017). We examined the microbiota in one early-phase batch and two aged batches of indigo fermentation fluid. The first batch (D1: aged 6 and 10 months) mainly consisted of the genera *Alkalibacterium*, *Amphibacillus*, and *Tissierellaceae* (**Figure 5**). The second batch (D2: aged 9, 11, and 14 months) mainly consisted of *Tissierellaceae*, *Proteinivoraceae*, and the genera *Anaerobacillus*, *Amphibacillus*, *Alkalibacterium*, and *Polygonibacillus* (Bacillaceae) (indigo-reducing bacteria, described below) (**Figure 5**). It can be assumed that *Anaerobacillus* spp. contain indigo-reducing bacteria based on their phylogenetic position adjacent to indigo-reducing bacteria and the Fe^3+^-reducing characteristic, as described above. Therefore, the two types of aged fermentation fluids evaluated in this study were probably primarily composed of indigo-reducing bacteria. The first batch mainly consisted of aerotolerant anaerobes, whereas a majority of the bacteria in the second batch was obligately anaerobic. Thus, there are several possible microbial communities that can perform indigo fermentation for long periods. Although these fermentation fluids remained in the indigo-reducing state for a long period, the microbiota changed dramatically in each batch. These successive changes in the microbiota involved in indigo reduction sustained the indigo-reducing state for this extended period.

**FIGURE 6:**
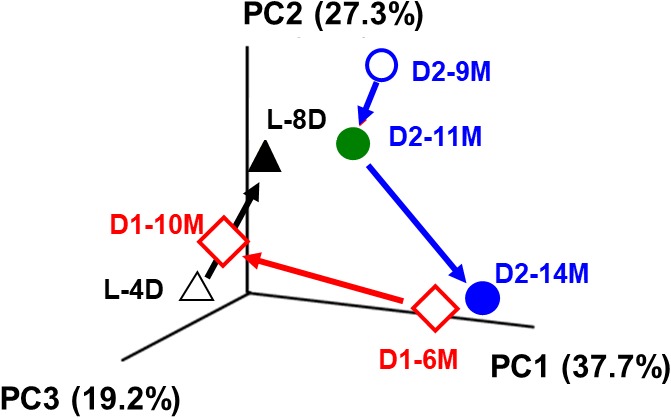
PCoA plot for the bacterial community of the indigo fermentation fluid. Analyzed samples are the initial (L-4D and L-8D) and aged fermentation phases (D1-6M, D1-10M, D2-9M, D2-11M, and D2-14M). L-4D, 4th-day sample, prepared in the laboratory; L-8D, 8th-day sample, prepared in the laboratory; D1-6M, 1st-batch sample, obtained from Date City (42°42′ N, 140°42′ E), aged 6 months; D1-10M, 1st-batch sample, obtained from Date City, aged 10 months; D2-9M, 2nd-batch sample, obtained from Date City, aged 9 months; D2-11M, 2nd-batch sample, obtained from Date City, aged 11 months; D2-14M: 2nd-batch sample, obtained from Date City, aged 14 months. This figure was cited from a figure in Okamoto et al. (2017). This reproduction was permitted by Springer-Nature.

We also analyzed the microbiota in the early fermentation period and used principal coordinate analysis (PCoA) to compare two batches maintained for a long period (Okamoto et al., 2017; **Figure 6**). Although the transition was slower in the aged fermentation fluid (batches D1 and D2) than in the fluid from the early fermentation period (L batch), substitution of the dominant indigo-reducing bacteria reflected the substantial changes that occur over 3 or 4 months in fermentation baths maintained for longer than 6 months (batches D1 and D2). Although the rates at which the changes occurred were dependent on fermentation conditions, the microbiota changed over the entire fermentation period under all the conditions. These changes may reflect the sustainability of the microbiota over a long period. It is expected that although the microbiota during the preliminary period of fermentation exhibited high flexibility, changes readily occurred in various directions, followed by the formation of a relatively stable state, due to relatively small changes in the 2 months between D2-9M and D2-14M (**Figure 6**). The stable state is characterized mainly by the genus *Anaerobacillus* and may contribute to the duration of fermentation. Over long maintenance periods, the resiliency of the microbiota and the proportion of indigo-reducing bacteria are expected to decrease, although no examples of such a state were detected in these analyses of the microbiota. In the case of indigo fermentation, in spite of the risk of bacterial contamination, substrates such as wheat bran are occasionally added, depending on the dyeing intensity. Therefore, the energy supply for indigo reduction remains high for a long period. In conclusion, the duration of sustained fermentation may be determined by the proportion of indigo-reducing bacteria and the stability of the microbiota, as observed in aged fermentation fluids.

**FIGURE 7:**
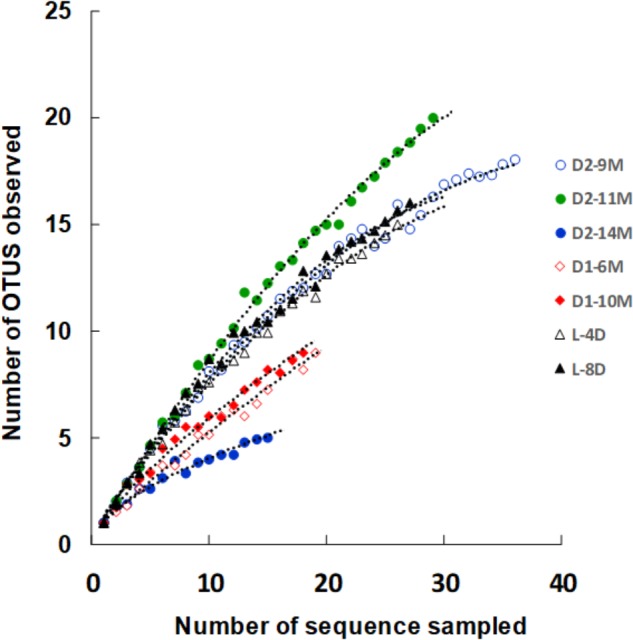
Rarefaction curve of OTUs defined by 3% sequence variation in samples. The indigo fermentation fluids in the initial (L-4D and L8D) and aged phases (D1-6M, D1-10M, D2-9M, D2-11M, and D2-14M) were analyzed. The curve depicts the diversity of each sample. Sample names are the same as those in **Figure 6**. OTUs, operational taxonomic units. This figure was reproduced from a figure in Okamoto et al. (2017). This reproduction was permitted by Springer-Nature.

Changes in the microbial diversity of the three different batches were estimated (**Figure 7**). Although distinct changes in the microbiota were observed over a short period in batch L, the corresponding bacterial diversity exhibited little variation. In addition, although distinct changes in the microbiota were observed over a long period in batch D1, there was little corresponding change observed in the diversity of the microbiota. This low diversity may explain why this batch was maintained for a longer than average duration. On the other hand, a distinct change in the diversity of the microbiota was observed in batch D2. An especially dramatic change was observed between 11 and 14 months of fermentation. One reason for the differences in the changes in diversity observed in the different batches may be the differences in the preparation and maintenance procedures. In fact, subtle differences in preparation procedures are thought to induce substantial differences in the microbiota and its diversity.

## Novel Procedure for the Isolation of Indigo-Reducing Bacteria

As described above, various indigo-reducing bacteria exist in the indigo fermentation liquor, and these bacteria often cannot be detected via culture-independent approaches. In addition, all the indigo-reducing bacterial species in various fermentation fluids and the different fermentation periods of these bacteria have yet to be identified. Until these bacteria are isolated and their indigo-reducing abilities are examined, it will not be possible to identify these species as indigo-reducing bacteria. Therefore, efficient procedures for the isolation of indigo-reducing bacteria are important not only for the accumulation of knowledge regarding indigo-reducing bacteria in fermentation fluid but also for interpreting the results of culture-independent approaches (especially the proportions of indigo-reducing genera or species). However, isolation of indigo-reducing bacteria is not always easy because conventional media are often unsuitable for slow-growing bacteria, which exist at low proportions. Hydrolysates of polysaccharides present during indigo fermentation and unknown substances in the fermentation material (i.e., *sukumo*) are appropriate candidates for promoting isolation of unknown indigo-reducing bacteria. In addition, the use of low nutrient levels to prevent the growth of rapidly growing bacteria will aid the isolation of slow-growing indigo-reducing bacteria. Wheat bran hydrolyzed by cellulase and *sukumo* hydrolyzed by cellulase have been used as novel media components for the isolation of new indigo-reducing bacteria. Media composed of each individual hydrolysate and mixtures thereof are prepared, and the sample-inoculated media is incubated under anaerobic conditions (Nishita et al., 2017). Indigo carmine has also been employed as an indicator of indigo reduction. Indigo fermentation fluid obtained from the indigo-dyeing craft center in Date City, Hokkaido, Japan, was inoculated onto prepared media, including conventional media. Media containing *sukumo* hydrolysate facilitated the isolation of novel *B. pseudofirmus*-related strains (later described as *P. indicireducens* gen. nov., sp. nov.), whereas media containing wheat bran hydrolysate facilitated the colonization of *Amphibacillus* spp. (including novel species, with lower than 98% sequence similarity of 16S rRNA sequences). Seven species (including two novel species) and six species (including three new species) of indigo-reducing bacteria were isolated using wheat bran hydrolysate-containing medium and medium containing both wheat bran and *sukumo* hydrolysate, respectively. These isolated species were more numerous than those in the conventional media. The media that were prepared for the first time in this study may also be useful for facilitating the isolation of bacteria other than indigo-reducing strains from indigo fermentation fluid samples. With this trial, we identified a previously isolated bacterium, *B. cohnii*, as an indigo-reducing bacterium (**Table 1**). This species is a facultative anaerobe and was isolated from all the tested media. Furthermore, new species of bacteria might exist in indigo fermentation mixtures prepared using different materials (e.g., different types of *sukumo*) or different preparation procedures. With the media used in this study, the isolation of even more species can be facilitated than via the procedures that use conventional media.

## Additional Indigo-Reducing Bacteria Isolated Using Novel Media

During the evaluation of the transitions of the microbiota and the trials aimed at isolation from new media, several novel strains were isolated and identified as novel taxa, including two new species belonging to new genera. These species likely appear late in the stable phase of indigo fermentation, approximately 5 months after the initiation of fermentation. Therefore, it is thought that some of these bacteria can be isolated from an indigo fermentation mixture older than 5 months using only specific media. On the other hand, some species of indigo-reducing bacteria can be isolated from indigo fermentation mixtures older than 5 months using ordinary media.

Strains In-9^T^ and D2-7 were isolated from 11-month-old fermentation fluid obtained from the craft center in Date City, Hokkaido, Japan (Okamoto et al., 2017). The obtained samples were inoculated onto indigo-carmine-containing conventional medium. The phylogenetic tree constructed from the sequences of these strains and those of related taxa suggested that strains In-9^T^ and D2-7 occupied distinct positions among the members of the genus *Bacillus* (strain In-9^T^ in **Figure 4**). In addition to their distinct positions in the phylogenetic tree, these strains could be discriminated from neighboring species or genera based on spore morphology and molecular species of menaquinone. Therefore, these strains represent a novel species within a novel genus, for which the name *Polygonibacillus indicireducens* gen. nov., sp. nov. was proposed (Hirota et al., 2016b; **Table 1**). This species was frequently detected in 11-month-old samples from the evaluated batch. Therefore, this species can be detected during only a limited period and only in certain batches. However, there is a possibility that this species contributes to indigo reduction as a minor constituent of the microbiota in various fermentation batches and various fermentation periods.

An aliquot of the sample was inoculated onto media containing *sukumo* hydrolysate and incubated under anaerobic conditions as described above. Thus, strains Bps-1^T^, Bps-2, and Bps-3 were isolated during a stable fermentation period from indigo fermentation fluid obtained from the indigo-dyeing craft center in Date City, Hokkaido, Japan. The phylogenetic tree constructed from the sequences of these strains and those of related taxa suggested that strains Bps-1^T^, Bps-2, and Bps-3 occupied distinct positions, based on 16S rRNA gene sequences, among the members of the genus *Bacillus* (strain Bps-1^T^ in **Figure 4**). In addition to their distinct position in the phylogenetic tree, these strains can be discriminated from neighboring species or genera based on their flagella and spore morphologies. Therefore, these strains represent a novel species within a novel genus, for which the name *P. indicireducens* gen. nov., sp. nov. was proposed (Hirota et al., 2017; **Table 1**). Although they were isolated from media containing *sukumo* hydrolysate, these strains do not require *sukumo* hydrolysate for growth. It is possible that components of *sukumo* hydrolysate support colony formation of these strains from the sample contained numerous bacteria.

During the above-described trials aimed at the isolation of indigo-reducing bacteria using various media, 13 strains were isolated (strain Bf-1^T^ in **Figure 4**). These strains were isolated from all the media used in the study (Nishita et al., 2017). Therefore, conventional media are applicable for the isolation of such strains. Among the 13 isolated strains, three strains, namely, Bf-1^T^, Bf-2, and Bf-4, were selected for taxonomic evaluation. Based on the polyphasic study, including phenotypic, chemotaxonomic, and genetic characterizations, these isolates represented a novel species, for which the name *Bacillus fermenti* sp. nov. was proposed (Hirota et al., 2018; **Table 1**). Furthermore, the isolates (obligate alkaliphiles) were clearly differentiated from other neighbors (neutralophilic or alkali-tolerant bacteria) in the maximum-likelihood phylogenetic tree derived from 16S rRNA gene sequences (**Figure 4**). The divergent lineage of the phylogenetic groups may reflect the selective pressures placed upon the organisms by the environmental conditions that these organisms have encountered, resulting in the evolution of an obligate alkaliphile that cannot grow at neutral pH. These findings suggest that these isolates evolved in a niche with a continuous high-alkaline environment, which would eliminate neutralophilic or alkali-tolerant bacteria.

## Conclusion and Perspectives

Rapid initiation of indigo reduction is an important concern in indigo fermentation using *sukumo*. If the desired transitional change in the microbiota occurs, the redox potential decreases rapidly on the 2nd day from the initiation of fermentation. On the 4th day, the abundances of obligately aerobic bacteria dramatically decrease, and aerotolerant and obligately anaerobic bacteria dominate the system, resulting in initiation of the staining of the soaked textile. If appropriate bacteria exist in the *sukumo*, the abundance of these desirable microorganisms increase under anaerobic alkaline conditions with appropriate materials and preparation and maintenance procedures. The transition of the microbiota is faster during the early stage of fermentation than during the stationary stage. This rate may be associated with the adaptability and stability of the microbiota at the early and stationary stages, respectively.

At the beginning of this study, we did not assume that many kinds of indigo-reducing bacteria existed in indigo fermentation fluid. In addition, we assumed that the microbiota profiles did not differ much among the various stages of indigo fermentation. By analysis of the transition of the microbiota, we found that indigo reduction can be performed by different microbial communities. In fact, it was observed that many species of bacteria could reduce indigo. Therefore, microbial communities with various profiles may exhibit indigo-reducing activity. Substitution of indigo-reducing bacteria may be an important factor for maintaining the indigo-reducing ability for an extended period.

Electron mediators such as quinone, which originate from plants or are produced by microorganisms, may exist in indigo fermentation fluid, especially in woad vat fermentation. However, there may be bacteria that can transfer electrons to extracellular substances without using electron mediators. There are reported cases of such bacteria (Logan, 2009), and these cases fall under three categories. For the first type of bacteria, the final electron acceptors are metal compounds instead of oxygen, and this process is called metal respiration. Electrons that are used for the production of ATP are discarded to extracellular metals, which act as electron sinks. The second type are bacteria that produce their own extracellular electron shuttles. Because the amounts of electron shuttle produced by most of these cases were not high enough for easy detection, there are few examples of such bacteria. There are examples of bacteria that produce flavin and extracellular cytochrome (Marsili et al., 2008; Smith et al., 2014). The third type is bacteria that produce electrically conductive pilus-like “nanowires” that extend outward from the electron-donating bacterial cells. This phenomenon has been reported in the case of *Shewanella oneidensis* (Gorby et al., 2006). It is considered that the mechanism of indigo reduction by indigo-reducing bacteria involves electrons, as byproducts of metabolism, being discarded by any of the methods described above under alkaline anaerobic condition.

There have not been many analyses of the microbiota present in natural fermentation systems. Traditional fermented foods include many examples of natural fermentation. However, fermentation in most of these cases occurs in acidic and salty environments, and it is difficult to manage the microbiota appropriately in nonselective conditions (i.e., conventional neutral conditions). There are few available examples of fermentation in alkaline environments. In indigo fermentation, high pH values and anaerobic conditions exert selective pressures on the microorganisms. This fermentation lasts for 6 months under conditions in which there are many chances for contamination. In addition, the result of indigo fermentation is distinct, and the properties of these fermentation systems can be easily and rapidly tested. The identified microbiota is not very complicated compared with those identified in conventional, natural, neutral environments, such as soil. Therefore, we conclude that indigo fermentation systems are suitable models for unsterilized and long-term natural fermentation. Elucidation of the mechanisms by which the microbiota exhibits resilience and persistence and of the interactions between constituent microorganisms in the microbiota will help improve not only the management of indigo fermentation but also maintenance methods for other anaerobic alkaline environments, such as Fe^3+^ reaction systems (Hobbie et al., 2012; Fuller et al., 2014) for producing electricity or halophilic alkaline natural fermentation systems for foods (Ouoba et al., 2010; Roth et al., 2011; Lucena-Padrós and Ruiz-Baba, 2016).

## Author Contributions

HM and IY designed the research. KA, KH, TO, and ZT performed the research. IY analyzed the data and wrote the paper.

## Conflict of Interest Statement

The authors declare that the research was conducted in the absence of any commercial or financial relationships that could be construed as a potential conflict of interest.
